# Interaction between CXCR4 and CCL20 Pathways Regulates Tumor Growth

**DOI:** 10.1371/journal.pone.0005125

**Published:** 2009-04-02

**Authors:** Katia Beider, Michal Abraham, Michal Begin, Hanna Wald, Ido D. Weiss, Ori Wald, Eli Pikarsky, Rinat Abramovitch, Evelyne Zeira, Eithan Galun, Arnon Nagler, Amnon Peled

**Affiliations:** 1 Goldyne Savad Institute of Gene Therapy, Hadassah Hebrew University Hospital, Jerusalem, Israel; 2 Department of Pathology, Hadassah Hebrew University Hospital, Jerusalem, Israel; 3 MRI Lab, HBRC, Hadassah Hebrew University Hospital, Jerusalem, Israel; 4 Bone Marrow Transplantation Department, Chaim Sheba Medical Center, Tel-Hashomer, Israel; University of Birmingham, United Kingdom

## Abstract

The chemokine receptor CXCR4 and its ligand CXCL12 is overexpressed in the majority of tumors and is critically involved in the development and metastasis of these tumors. CXCR4 is expressed in malignant tumor cells whereas its ligand SDF-1 (CXCL12) is expressed mainly by cancer associated fibroblasts (CAF). Similarly to CXCR4, the chemokine CCL20 is overexpressed in variety of tumors; however its role and regulation in tumors is not fully clear. Here, we show that the chemokine receptor CXCR4 stimulates the production of the chemokine CCL20 and that CCL20 stimulates the proliferation and adhesion to collagen of various tumor cells. Furthermore, overexpression of CCL20 in tumor cells promotes growth and adhesion in vitro and increased tumor growth and invasiveness in vivo. Moreover, neutralizing antibodies to CCL20 inhibit the in vivo growth of tumors that either overexpress CXCR4 or CCL20 or naturally express CCL20. These results reveal a role for CCL20 in CXCR4-dependent and -independent tumor growth and suggest a therapeutic potential for CCL20 and CCR6 antagonists in the treatment of CXCR4- and CCL20-dependent malignancies.

## Introduction

Chemokines, a family of small (5–20 kDa) pro-inflammatory cytokines, are primarily responsible for the directional migration, or chemotaxis, of lymphocytes to specific lymphoid tissues, and the recruitment of leukocytes to the sites of infection or tissue damage. However, chemokines are also implicated in other biological events including angiogenesis, angiostasis, embryogenesis, hematopoiesis, lymphopoiesis, and HIV pathogenesis [1]. More recently, it has been established that cancer cells exploit signaling through chemokine receptors via several key steps involved in initiation and progression of primary and metastatic cancer. Different types of cancers express different chemokine receptors [2], [3], however, only the chemokine receptor CXCR4 appears to be expressed by the majority of cancer types. Tumor cells from at least 23 different types of cancers of epithelial, mesenchymal and haematopoietic origin express CXCR4 [4]. Moreover, CXCR4 expression was found to be increased in several malignancies including gliomas, breast tumors, certain leukemia cell lines, uterine cancer, Burkitt's lymphoma, neuroblastomas, and pancreatic cancer [5], [6], [7], [8], [9], [10]. CXCR4 was also found to play a critical role in the progression and development of various tumors including breast, prostate and clear cell renal carcinoma [6], [11], [12], [13]. The importance of the CXCR4/CXCL12 pathway in tumor development was further demonstrated by neutralizing the interaction between CXCL12 and CXCR4. [6], [14], [15], [16]. The mechanisms by which CXCR4 regulate tumor progression are yet not clear. Our recent results provide a novel mechanism for CXCR4-mediated tumor growth and metastasis and establish a functional link between CXCR4/CXCL12 and CCR6/CCL20 pathways in tumor development.

## Results

### CXCR4 up-regulates CCL20 mRNA and protein expression in prostate cancer cells

**Figure 1:**
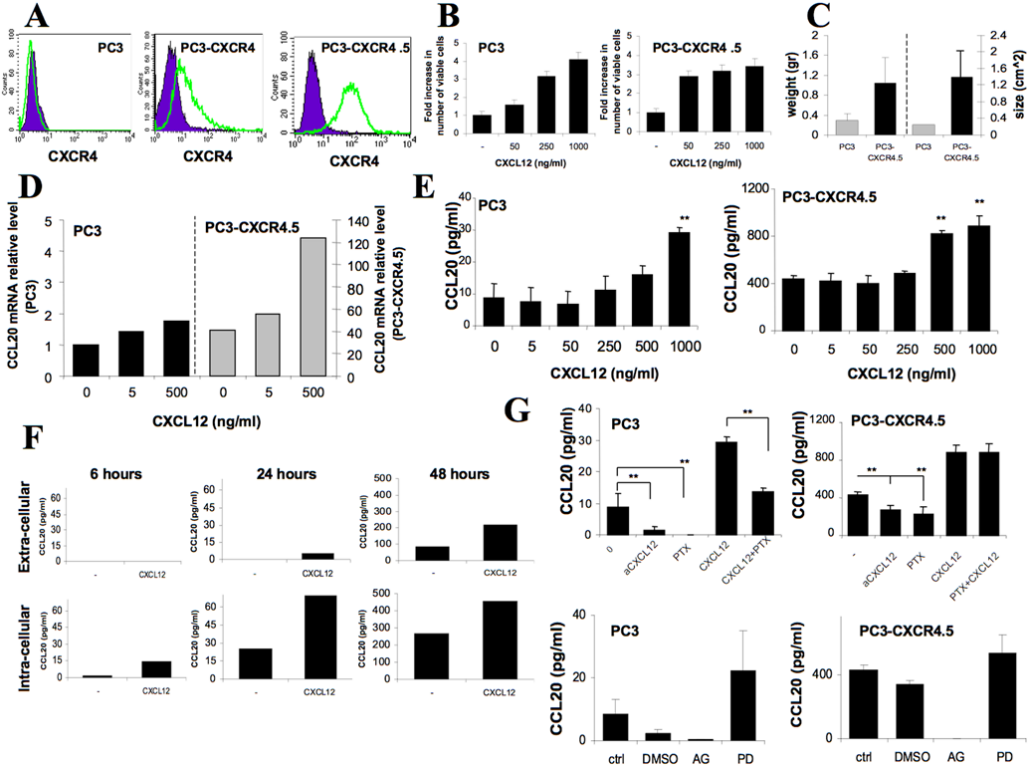
CXCR4 up-regulates CCL20 production by prostate cancer cell line PC3. (A–C) Wild-type PC3 cells, CXCR4-transduced PC3 cell line and single cell clone with stable overexpression of CXCR4 (PC3-CXCR4.5) were stained for the control (IgG2a-PE, purple) and CXCR4 antibodies (IgG2a-12G5, green) and analyzed using FACS. PC3 and PC3-CXCR4.5 cells were stimulated with CXCL12 at concentrations of 50, 250 and 1,000 ng/ml for 48 hours, harvested and viable cells were counted using PI staining and FACS analysis. PC3 and PC3-CXCR4.5 cells were (5×10^6^/mice) were injected subcutaneously into SCID/beige mice. 60 days following the injection, animals were sacrificed, tumor size (cm^2^) and tumor weight (g) were measured. Data is presented as mean±SE from 5 mice. (D) PC3 and PC3-CXCR4.5 cells were stimulated with CXCL12 at concentrations 5 and 500 ng/ml for 24 hours, total RNA was extracted, reverse-transcribed and subjected to quantitative PCR for CCL20. PCR analysis was carried out in triplicates. (E) PC3 and PC3-CXCR4.5 cells were stimulated with various concentrations of CXCL12 (5, 50, 25, 500, and 1,000 ng/ml) for 48 hours and CCL20 secretion was assessed by ELISA. The results represent the average of triplicates±SD (** *P*<0.05). (F) PC3-CXCR4.5 cells were incubated with CXCL12 at concentration of 500 ng/ml. At the indicated time points CCL20 was assessed in extra-cellular (culture medium) and intra-cellular (whole cell lysate) fractions using ELISA method. (G) In order to inhibit CXCR4 signaling, PC3 and PC3-CXCR4.5 cells were cultured with anti-CXCL12 antibodies or pertussis toxin alone or in combination with CXCL12 during 48 hours, and CCL20 secretion was assessed by ELISA. CCL20 secretion in PC3 and PC3-CXCR4.5 cells was also inhibited using JAK-2 inhibitor AG-490 at 1 µm/ml. and the MEK inhibitor- PD98059 (20 µM). The results represent the average of triplicates±SD (** *P*<0.05).

In our previous work, we provided the evidence that the CXCR4 receptor promotes prostate tumor growth, invasion and vascularization [17]. In this study, we generated single-cell clones of CXCR4-transduced PC3 cells with high and stable levels of CXCR4 expression (Fig. 1A). CXCR4-transduced PC3 cells with high and stable levels of CXCR4 expression demonstrated a high proliferation rate *in vitro* in response to stimulation with CXCL12, and increased sensitivity to low concentrations of CXCL12, compared to wild-type PC3 cells (Fig. 1A). Moreover, mice injected subcutaneously with these cells developed bigger tumors compared to mice injected with wild-type PC3 cells, similarly to our previous results (Fig. 1B,C).

In order to identify genes that are regulated by CXCR4 and contribute to the aggressive phenotype of CXCR4-expressing prostate cancer, we compared the expression pattern of various chemokines between CXCL12-stimulated and non-stimulated PC3-CXCR4.5 cells. We found an increase in expression of the chemokine CCL20 following stimulation with CXCL12. To further study the possible role of CXCR4 in the regulation of CCL20 expression, we evaluated the mRNA expression of CCL20 in prostate cancer cells with low and high CXCR4 expression using real-time RT-PCR. We found a 41-fold increase in CCL20 mRNA in cells with high CXCR4 expression compared to the wild-type PC3 cells. Moreover, stimulation of both PC3 and PC3-CXCR4.5 cells with CXCL12, the ligand of CXCR4, increased CCL20 mRNA levels in a dose-dependent manner (Fig. 1D). Next, we assessed whether the constitutive and regulated expression of CCL20 mRNA was paralleled by protein production, using an ELISA assay to CCL20. Corresponding with PCR results, CXCR4 over-expression in PC3 cells increased the constitutive levels of CCL20 secretion from 8.6±4.5 pg/ml in PC3 cells to 433±30 pg/ml in PC3-CXCR4.5 cells (Fig. 1E). Moreover, the stimulation of both parental PC3 and PC3-CXCR4.5 cells with CXCL12 increased the secretion of CCL20 in a dose-dependent manner (Fig. 1E). To determine the pattern of CCL20 release, we tested the protein levels of CCL20 in a culture medium and in lysed cells following different periods of incubation with CXCL12. PC3-CXCR4.5 cells were incubated with CXCL12 500 ng/ml for 6, 24 or 48 hours; culture medium was collected and the cells were lysed with lysis buffer containing 1% NP-40. Stimulation with CXCL12 increased the intracellular levels of CCL20 following 6 hours of incubation, however there was no release of CCL20 to the culture medium at this time point. Following 24 hours of incubation, intra-cellular levels of CCL20 in non-stimulated and in CXCL12-stimulated cells were increased, as well the secreted CCL20 was detected in the culture medium of CXCL12-stimulated PC3-CXCR4.5 cells (no secretion in non-treated cells versus 4.9 pg/ml of CCL20 in CXCL12-treated cells). After 48 hours of incubation, the levels of intra-cellular CCL20 were significantly elevated and higher CCL20 production was observed in CXCL12-stimulated cells, in addition, the secretion of CCL20 was increased. Both synthesized and secreted CCL20 levels increased in response to CXCL12 stimulation, and the cumulative pattern of synthesis and release was observed over time (Fig. 1F).

In order to find whether PC3 and PC3-CXCR4.5 cells secrete CCL20 in a CXCR4/CXCL12 dependent manner, we treated the cells with antibodies to CXCL12 and PTX. In PC3 cells, treatment with neutralizing antibodies against CXCL12 or with PTX (alone or in combination with CXCL12) effectively inhibited the secretion of CCL20. Surprisingly, in PC3-CXCR4.5 cells, anti-CXCL12 antibodies and PTX only partially inhibited the secretion of CCL20 whereas PTX failed to inhibit CXCL12- dependent increase of CCL20 (Fig. 1G).

**Figure 2:**
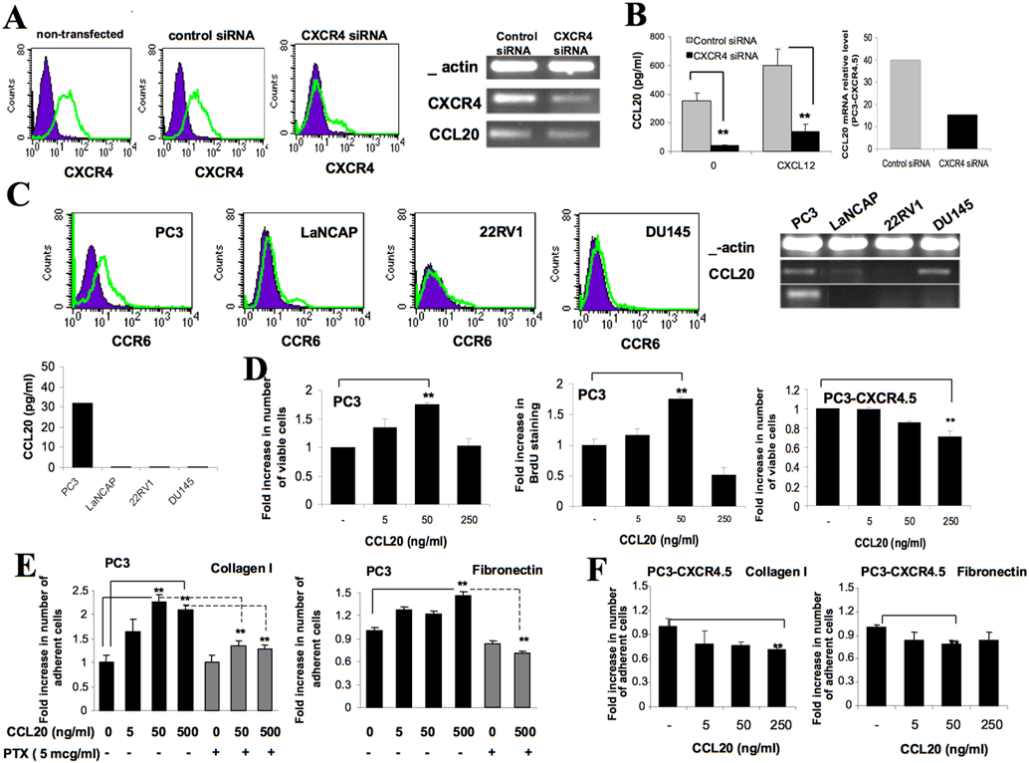
Regulation of CCL20 expression and function. (A–B) CXCR4 expression levels of PC3-CXCR4.5 cells 48 hours following the transfection with control non-specific siRNA and specific anti-CXCR4 siRNA. The cells were stained for the control and CXCR4 antibodies and evaluated by FACS. Semi-quantitative RT-PCR analysis of CXCR4 and CCL20 mRNA of the siRNA-transfected PC3-CXCR4.5 cells 48 hours following the transfection. β-actin confirmed comparable loading of RT-PCR products in each lane. Control and CXCR4 siRNA-transfected PC3-CXCR4.5 cells at 48 hours post-transfection were incubated with CXCL12 500 ng/ml for an additional 48 hours. CCL20 secretion was assessed by ELISA. The results represent the average of triplicates±SD (** *P*<0.05). (C) CCR6 expression in prostate cancer cell lines PC3, LaNCAP, 22Rv1 and DU145 was evaluated by FACS and PCR whereas CCL20 expresion was evaluated by PCR and ELISA assays. Purple line represents mouse IgG control antibody, green line represents staining with CCR6 monoclonal antibody. (D) PC3 and PC3-CXCR4 cells were incubated with various concentration of CCL20 for 6 days. Following 3 days of incubation, the medium with or without CCL20 was renewed. On day 6, the cells were harvested and viable cells were counted using PI staining and FACS analysis. In addition, in order to determine proliferation of PC3 cells, the cells were labeled with BrdU (10 µM) during the last 16 hours of incubation and processed for BrdU detection using specific anti-BrdU FITC-conjugated antibody and FACS analysis. Data is presented as mean±SD from triplicates (** *P*<0.05). Data is representative of two separate experiments. (E–F) PC3 and PC3-CXCR4.5 cells either untreated or treated with various concentrations of CCL20 were placed on collagen I- or fibronectin-coated plates (10 µg/ml) for 30 minutes. Non-adherent cells were washed twice with cold PBS. Adherent cells were collected in 300 µl FACS buffer with 5 mM EDTA and counted by FACS. Data is presented as mean±SD from triplicates (** *P*<0.05). In addition, PC3 cells (E) that demonstrated increased adhesion to collagen I and fibronectin in response to stimulation with CCL20, were co-incubated with CCL20 and PTX (100 ng/ml) and were allowed to adhere to collagen I- and fibronectin-coated plates.

CCL20 production from PC3 cells is dependent on CXCR4/CXCL12 interaction; however, it was not clear whether the expression and secretion of CCL20 from PC3-CXCR4.5 cells was dependent only on CXCR4. In order to determine the role of CXCR4 in the regulation of CCL20 production, we silenced CXCR4 by RNA interference in PC3-CXCR4.5 - prostate cancer cells with high CXCR4 expression. PC3-CXCR4.5 cells were transfected with Dicer-substrate anti-CXCR4 mRNA specific siRNA. The efficacy of CXCR4 silencing was assessed 48 hours post-transfection. We achieved over 90% reduction in CXCR4 cell surface expression (Fig. 2A) and 75% reduction in mRNA levels (Fig. 2A) using siRNA to CXCR4 RNA. Next, we sought to determine whether the reduction in CXCR4 might inhibit CCL20 production in PC3-CXCR4.5 cells. Forty eight hours following transfection with siRNA, PC3-CXCR4.5 cells were re-seeded and stimulated with CXCL12 for an additional 48 hours. CCL20 secretion levels in culture medium of the cells transfected with either nonspecific control siRNA or the CXCR4 siRNA were measured using ELISA assay. Silencing of CXCR4 significantly reduced both constitutive and CXCL12-stimulated levels of CCL20 production. In non-stimulated cells, the secretion of CCL20 was decreased from 350±56 pg/ml in control cell to 42±2.8 pg/ml in cells with CXCR4 siRNA (p<0.0054). In CXCL12-stimulated cells, the secretion of CCL20 was decreased from 600±113 pg/ml in control cells to 137.5±53 pg/ml in cells with CXCR4 siRNA (p<0.0035, Fig. 2A). In addition, we tested the effect of CXCR4 silencing on CCL20 mRNA level using real-time RT-PCR. Silencing of CXCR4 resulted in a 3-fold decrease in CCL20 mRNA level in PC3-CXCR4.5 cells (Fig. 2B).

CCL20 expression and secretion in the parental PC3 and PC3-CXCR4.5 cells that overexpress CXCR4 are regulated by CXCR4 (Fig. 1G and Fig. 2A). PC3 cells express low levels of CXCR4 and secrete low levels of CCL20, whereas PC3-CXCR4.5 cells express high levels of CXCR4 and ten-fold higher levels of CCL20. In both cell lines, treatment with PTX or anti CXCL12 blocked CCL20 secretion; however, the inhibitory effect of PTX or anti CXCL12 was much more evident in the parental cells. Similarly, the effect of PTX on CXCL12-induced secretion of CCL20 was partial, albeit significant only in the parental PC3 cells. The differences between these cells may be the result of a more robust activation of diverse signaling pathways activated by CXCR4 and the use of insensitive Gα chain used by CXCR4. Various signal transduction pathways have been implicated through the association of CXCR4 receptors with the guanine nucleotide binding protein (G-proteins). For example, stimulation of CXCR4 transfectants by CXCL12 results in increased phosphorylation of focal adhesion components, including the related adhesion focal tyrosine kinase (RAFTK/Pyk2), Crk, and paxillin. CXCL12 induced activation of the p44/42 MAP kinases (Erk 1 and 2), PI3 kinase, and 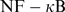 [18], [19], [20]. Moreover, a short time following CXCL12 activation, the CXCR4 receptor becomes tyrosine phosphorylated through the activation and association with the receptor of JAK2 and JAK3 kinases [21], [22]. Several STAT transcriptional factors, including STAT1, -2, -3, and -5, but not STAT4 or -6, are associated with CXCR4 activation, concurring with the role assigned to the JAK tyrosine kinases in transducing signals from hematopoietic growth factor receptors. Tyrosine phosphorylation of two other members of the chemokine receptor family, CCR2 and CCR5, in response to their respective ligands (MCP-1 and RANTES) and the critical role of the JAK/STAT pathway activation in later chemokine signaling events were also documented. The activation in T cells of different STATs by the chemokines RANTES and 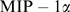 has also been reported. Tyrosine phosphorylation is not a unique feature of chemokine receptors, since other GPCRs also activate STAT transcription factors. Interestingly, JAK/STAT pathway activation by chemokines and their receptors is not always blocked by PTX pretreatment, indicating a G-protein-independent pathway. To address the importance of JAK/STAT and MEK activation on the secretion of CCL20, we treated the PC3 and PC3-CXCR4.5 cells with JAK-2 inhibitor AG-490 (Cat. 658401; Calbiochem) at 1 µm/ml. and the MEK inhibitor- PD98059 (20 µM) (Calbiochem, San Diego, CA) PD. In both cell lines, the JAK-2 inhibitor AG-490 totally abrogated CCL20 secretion, whereas the MEK inhibitor- PD98059 slightly increased the levels of CCL20. DMSO used for dissolving PD or AG-490 had an inhibitory effect on CCL20 secretion, and more significantly on the parental PC3 cells (Fig. 1G). These results may suggest a role for the JAK/STAT pathway in the CXCL12-induced secretion of CCL20.

The increased levels of CCL20 associated with increased levels of CXCR4 do not correlate with the CXCL12 low levels secreted by PC3 or PC3-CXCR4.5 cells and cannot be blocked by anti CXCL12 when added to PC3-CXCR4.5 cell cultures (Fig. 1G). One possibility to explain such a phenomenon is the presence of a CXCR4 ligand other than CXCL12. Although chemokines typically display a high degree of receptor promiscuity, CXCR4 was (until recently) thought to bind only to CXCL12. However, recently, it was demonstrated that the macrophage migration inhibitory factor (MIF) can compete with the recognized ligand for CXCL12 for binding to CXCR4 [23]. CXCR4 was shown to be homo-oligomerized by several experimental systems. It is possible that CXCR4 oligomerization may lead to autonomous signaling which is mediated through JAK/ STAT signaling.

To better understand the role of CCL20 in prostate cancer development, we characterized the expression of CCL20 and its receptor CCR6 in human prostate cell lines PC3, LNCaP, 22RV1 and DU145. We first examined CCR6 receptor surface and mRNA expression levels in these four cell lines. RT-PCR analysis and FACS analysis demonstrated that only PC3 cell line expressed CCR6 receptor at mRNA level and on the cell surface (Fig. 2C). Next, we performed ELISA experiments to determine the secretion levels of CCL20 chemokine. Among the four prostate cancer cell lines studied, only PC3 cells secreted detectable levels of CCL20 into the culture supernatant during the 48 hours incubation (Fig. 2C). However, in addition to PC3 cells, the mRNA expression of CCL20 was demonstrable in DU145 cells and at a very low level also in LANCaP cells (Fig. 2C). Since PC3 cells co-expressed the CCR6 receptor and its ligand CCL20, we focused our in vitro and in vivo experimental work on PC3 cells.

### CCL20 promotes the growth and adhesion of CCR6-expressing tumor cells in vitro

We hypothesized that the CCR6-CCL20 axis may be involved in tumor progression via paracrine and/or autocrine mechanisms. To assess biological behavior resulting from CCL20-mediated activation in PC3 cells, we studied the effect of CCL20 on the growth and survival of PC3 cells in culture. PC3 cells were treated with various concentrations of CCL20. The number of viable cells following six days of incubation was detected using propidium iodide (PI) and FACS analysis. Treatment of cells with CCL20 increased the number of viable PC3 cells in the culture at a concentration of 5 ng/ml (1.35-fold increase, p<0.04) and at a concentration of 50 ng/ml (1.75-fold increase, p<0.0004); whereas at the highest concentration of CCL20, 250 ng/ml, we found no change in the number of viable cells compared to control non-stimulated PC3 cells (Fig. 2D). To further verify these results, CCL20-treated cells were loaded with 5-bromo-2-deoxyuridine (BrdU). Cell proliferation was tested by staining for BrdU incorporation using specific anti-BrdU antibody and FACS analysis. Consistent with previous results, CCL20 induced the incorporation of BrdU in the cells at a concentration of 5 and 50 ng/ml (1.76-fold increase, p<0.0002), whereas treating the cells with higher concentration of CCL20 did not change the level of BrdU incorporation (Fig. 2D). In accordance with the previous results, we found that PC3-CXCR4.5 cells, which constitutively express high levels of CCL20, where inhibited in their growth when treated with increasing concentrations of CCL20 (Fig. 2D). Concentration of 250 ng/ml even decreased the number of PC3-CXCR4.5 viable cells (p<0.006).

Adhesion of cancer cells to extracellular matrix (ECM) components is a step that is associated with tumor seeding, invasion and spreading. In order to investigate the effect of CCL20 on PC3 cell adhesion to the ECM proteins, we tested the adhesion of PC3 cells to the fibronectin and collagen I in response to increasing concentrations of CCL20. As shown in Fig. 2E, elevated doses of CCL20 slightly increased the adhesion of cells to fibronectin (500 ng/ml of CCL20 promoted 1.45-fold increase, p<0.01), and significantly increased the adhesion of cells to collagen type I in a dose-dependent manner (Fig. 2E). Upon activation by CCL20 at concentration of 50 ng/ml PC3, adhesion to collagen I was 2.2-fold elevated (p<0.001); concentration of 500 ng/ml caused a 2.09-fold increase in adhesion (p<0.003). Treatment with pertussis toxin (PTX) prevented the CCL20-induced increase in PC3 cell adhesion to fibronectin (p<0.0009) and collagen I (p<0.002) (Fig. 2E). In contrast to PC3 cells , adhesion of CCL20-producing PC3-CXCR4.5 cells to collagen I and fibronectin was slightly decreased following stimulation with increased doses of CCL20 (Fig. 2F).

The exposure of chemokine receptors to high concentrations of chemokines often results in the rapid attenuation of receptor responsiveness and a reduced biological response. This process termed ‘desensitization’ is the consequence of a combination of different mechanisms. These mechanisms include receptor phosphorylation followed by uncoupling of the receptor from heterotrimeric G proteins and internalization of cell surface receptors to intracellular endocytic vesicles [24]. It is therefore possible that at high levels of CCL20, the chemokine receptor CCR6 is desensitized due to internalization and/or phosphorylation [25]. Indeed preliminary data suggest that high levels of CCL20 can stimulate the internalization of CCR6.

In addition to PC3 cells, we tested the effect of CCL20 activation on biological function of other human prostate cancer cell lines - LNCaP, 22RV1 and DU145 cells. *In vitro* proliferation and adhesion of the cells to collagen were tested in the absence or presence of different concentrations of CCL20. In agreement with CCR6 expression pattern, LNCaP, 22RV1 and DU145 cells that do not express CCR6, did not respond to CCL20 stimulation and no increase in proliferation or adhesion was observed (data not shown).

We therefore concluded that CCL20 can stimulate both PC3 cell proliferation and adhesion to collagen type I in a dose dependent manner.

**Figure 3:**
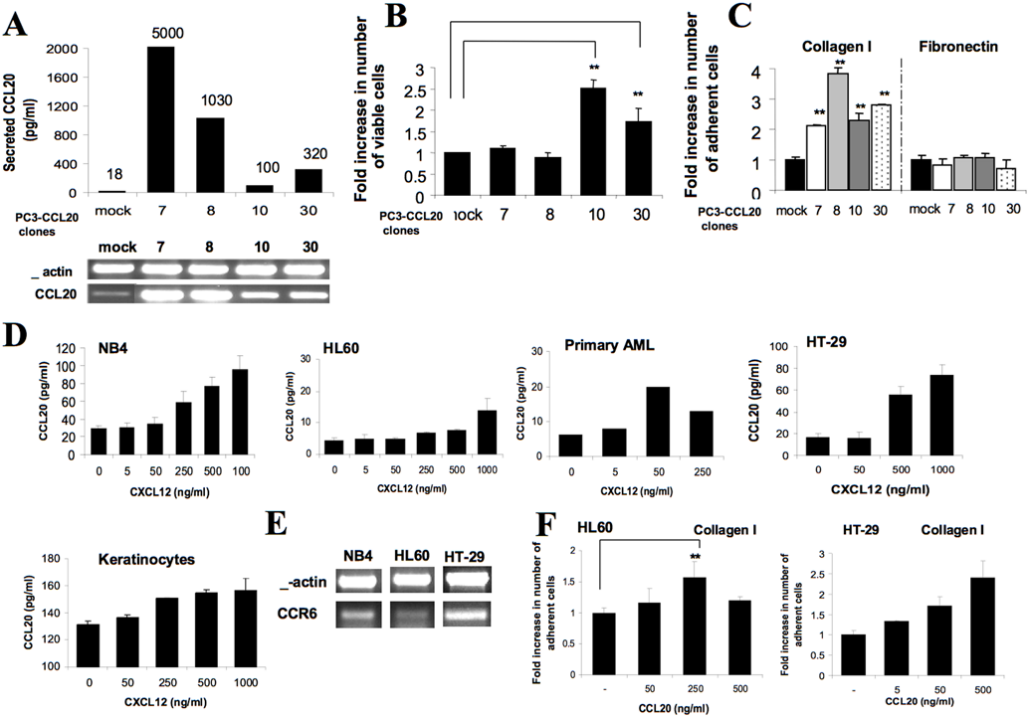
Regulation of CCL20 expression and function in various tumor cells. (A) CCL20 mRNA and protein expression in CCL20-transfected PC3 single-cell clones tested by semi-quantitative RT-PCR and ELISA. (B) PC3-CCL20 clones were seeded at 2×10^4^ cells/1 ml per well into a 24-well plate and incubated for 6 days. On day 6, the cells were harvested and viable cells were counted using PI staining and FACS analysis. Data is presented as mean±SD from triplicates (** *P*<0.05). (C) PC3-CCL20 single-cell clones were grown to confluence, harvested, resuspended in their conditioned culture medium and allowed to adhere to collagen I- and fibronectin-coated plates for 30 minutes. Non-adherent cells were washed, and adherent cells were collected in 300 µl FACS buffer with 5 mM EDTA and counted by FACS. Data is presented as mean±SD from triplicates (** *P*<0.05). (D) Leukemic cell lines NB4 and HL60, primary human leukemic blasts, HT-29 cells, and normal human keratinocytes were incubated with various concentrations of CXCL12 for 48 hours. CCL20 secretion to culture medium was assessed using ELISA method. (E) CCR6 mRNA expression in leukemic cell lines NB4 and HL60 and colon cancer HT-29 cells assessed by semi-quantitative RT-PCR. β-actin confirmed comparable loading of RT-PCR products in each lane. (F) HL60 and HT-29 cells either untreated or treated with various concentrations of CCL20 were placed on collagen I coated plates (10 µg/ml) for 30 minutes. Non-adherent cells were washed twice with cold PBS. Adherent cells were collected in 300 µl FACS buffer with 5 mM EDTA and counted by FACS. Data is presented as mean±SD from triplicates (** *P*<0.05). Data is representative of three separate experiments.

In order to further determine the involvement of CCL20 in cancer development in vitro, we introduced the CCL20 gene into prostate PC3 cells which express the CCR6 receptor. PC3 cells were stably transfected with vector encoding CCL20, and different clones overexpressing CCL20 were obtained. The levels of CCL20 were quantified using PCR and ELISA assay (Fig. 3A). Clone numbers 7 and 8 demonstrated the highest levels of CCL20 secretion (5,000 and 1,030 pg/ml, respectively) whereas clone numbers 10 and 30 showed moderate levels of CCL20 secretion (100 and 320 pg/ml, respectively).

Next, we tested the growth of PC3 clones that overexpress CCL20. The number of viable cells following six days of incubation was detected using PI and FACS analysis. PC3-CCL20 clones that secrete moderate levels of CCL20, PC3-CCL20.10 and PC3-CCL20.30, demonstrated 1.7-fold and 2.5-fold increases in the number of viable cells in culture, respectively (Fig. 3B). The growth of PC3-CCL20 clones that secrete high levels of CCL20 (PC3-CCL20.7 and PC3-CCL20.8) was similar to parental control PC3 cells (Fig. 3B). This may suggest that autocrine secretion of CCL20 may drive tumor growth.

To further explore the effect of CCL20 overexpression on prostate cancer cell behavior, we assessed the adhesion of PC3 clones that overexpress CCL20 to ECM proteins fibronectin and collagen I. CCL20-overexpressing PC3 cells were grown to confluence, harvested, and allowed to adhere to fibronectin or collagen I-coated plates. Over-expression of CCL20 significantly increased the adhesion of all four CCL20-expressing clones to the collagen I. Comparing to mock-transfected PC3 cells, clones 7, 8, 10 and 30 demonstrated 2.1-fold (p<0.04), 3.8-fold (p<0.01), 2.2-fold (p<0.03) and 2.8-fold (p<0.004) increases in their adhesion to collagen I, respectively (Fig. 3C). No significant change was observed in the adhesion of these cells to the fibronectin (data not shown).

CXCR4 and CCL20 expression has been described in a variety of human neoplasms, including colorectal, lung, pancreatic and breast human adenocarcinomas, malignant glioma, leukemia, lymphoma and melanoma as well as by normal keratinocytes. We therefore next tested by ELISA the expression of CCL20 in a range of human cancer cell lines. We found that in addition to PC3 cells, CCL20 was secreted by promyelocytic leukemia (APL) cell lines, NB4 and HL60, by primary blasts of patients with acute myelocytic leukemia, human HT-29 colon carcinoma cells as well as and by normal keratinocytes (Fig. 3D). Furthermore, in NB4, HL60, primary AML blasts, HT-29 cells, and normal keratinocytes, the secretion of CCL20 was increased following stimulation with CXCL12, in a dose-dependent manner (Fig. 3D). Next, we characterized the expression of the CXCR4 receptor on NB4, HL60 cells, AML blasts and HT-29 cells. Leukemic lines NB4 and HL60, primary human AML blasts and HT-29 cells demonstrated high cell-surface expression levels of the CXCR4 receptor (data not shown). These results suggest a more general role for CXCR4 in regulating CCL20 expression in various cancer cells.

To confirm the role of CCL20 in autocrine stimulation of cancer cells from a different origin, we tested the expression of CCR6 in CCL20-secreting NB4, HL60, and HT-29 cells. We found that NB4, HL60, and HT-29 cells expressed CCR6 at the mRNA level (Fig. 3E), however HL60 possessed higher levels of cell-surface CCR6 than NB4 and HT-29 cells (data not shown). Next, we tested the effect of CCL20 stimulation on NB4, HL60, and HT-29 cell proliferation and adhesion to the ECM components, collagen I and fibronectin. We found a 1.6-fold increase in adhesion of HL60 cells to collagen I upon simulation with CCL20 250 ng/ml (p<0.006) and a 2-fold increase in adhesion to fibronectin upon stimulation with 500 ng/ml (p<0.015) (Fig. 3F, data not shown). Stimulation of HT-29 resulted in a dose-dependent increased adhesion to collagen type I, but not fibronectin (Fig. 3F, data not shown). In contrast to HL60 and HT-29, NB4 cells that expressed low surface level of CCR6 did not proliferate or adhere to fibronectin or collagen in response to CCL20 (data not shown).

### Overexpression of CCL20 increases the growth, invasion and vascularization of PC3 cells in vivo

**Figure 4:**
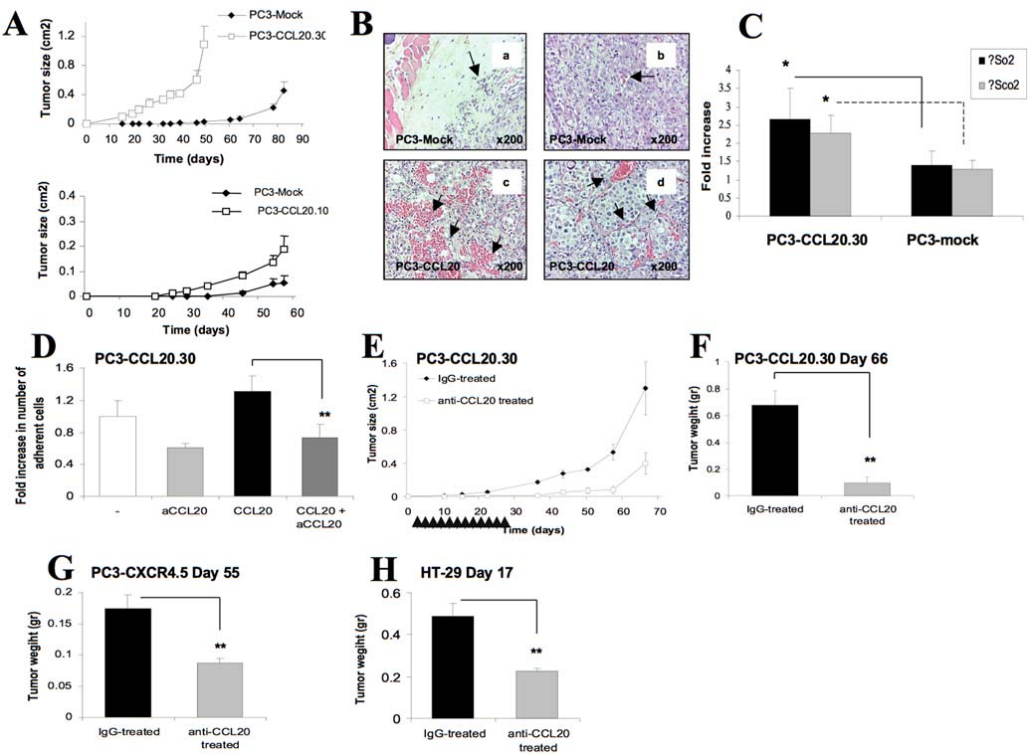
CCL20 regulates CXCR4 dependent and independent growth of tumor cells. (A) Effect of CCL20 stable expression on prostate tumor growth. PC3-CCL20.30, PC3-CCL20.10 and PC3-mock transfected cells (5×10^6^/mouse) were injected subcutaneously into SCID/beige mice. 48 days or 75 days following the injection, animals were sacrificed, and xenograft tumors generated by PC3-mock and PC3-CCL20.30 injected cells were purified . Tumor size (cm^2^) was measured twice a week using caliper. Results are representative of three independent experiments with five mice in each group. Data is presented as mean±SE from five mice. (B) H&E staining of paraffin-embedded tumor tissue sections derived from PC3-mock and PC3-CCL20.30 tumors on day 48. Black arrows signify non-invasive borders of PC3-mock tumor (B,a), small blood vessel in PC3-mock tumor (B,b), aberrant blood vessels in PC3-CCL20.30 tumor (B, c,d), original magnification of ×200 is shown. (C) Vessel functionality (Δ*S*o_2_) was measured by fMRI. Functionality of the vasculature was tested during inhalation of air-CO_2_ and carbogen (95% oxygen+5% CO_2_) in mice implanted with PC3-mock cells or with PC3-CCL20.30 cells. Δ*S*o_2_ values from PC3-mock cells and PC3-CCL20.30 are shown. The mean±SD values of Δ*S*o_2_ were calculated from whole tumor, and normalized to contra-lateral muscle, pooling data from 9 mice from the PC3-CCL20.30 group and 5 mice from the PC3-mock group (four slices/mouse; p<0.001). (D) Adhesion of PC3-CCL20.30 cells to collagen I. PC3-CCL20.30 cells either umstimulated or stimulated with 50 ng/ml of CCL20 with or without co-incubation with neutralizing anti-CCL20 antibodies (10 µg/ml) were placed on collagen I-coated plates (10 µg/ml) for 30 minutes. Non-adherent cells were washed twice with cold PBS. Adherent cells were collected in 300 µl FACS buffer with 5 mM EDTA and counted by FACS. Data is presented as mean±SD from triplicates (** *P*<0.016). (E) PC3-CCL20.30 cells (5×10^6^/mouse) were injected subcutaneously into SCID/beige mice. Twenty-four hours after the cell injection, mice started to get subcutaneous injections of anti-human CCL20 antibodies or isotype control antibodies, 20 µg of antibody per injection, three times a week, during four weeks. Tumor size (cm^2^) was measured once a week using caliper. Results are representative of two independent experiments with ten mice in each group. Data is presented as mean±SE from ten mice. (F) On day 64 following the cell injection, the experiment was terminated, animals were sacrificed; subcutaneous tumors were removed, measured and weighted. Data is presented as mean±SE from ten mice in each group (** *P*<0.0002). (G) PC3-CXCR4.5 cells (5×10^6^/mouse) were injected subcutaneously into SCID/beige mice. Twenty-four hours after the cell injection, mice started to get subcutaneous injections of anti-human CCL20 antibodies or isotype control antibodies, 20 µg of antibody per injection, three times a week, during four weeks. Tumor size (cm^2^) was measured once a week using caliper. On day 55 following cell injection, the experiment was terminated; animals were sacrificed, subcutaneous tumors were removed, measured and weighted. Data is presented as mean±SE from ten mice in each group (** *P*<0.0027). (H) HT-29 cells (2×10^6^/mouse) were injected subcutaneously into nude mice. Twenty-four hours after cell injection, mice started to get subcutaneous injections of anti-human CCL20 antibodies or isotype control antibodies, 20 µg of antibody per injection, five times a week, during two weeks. On day 17 following cell injection, the experiment was terminated, animals were sacrificed; subcutaneous tumors were removed, measured and weighted. Data is presented as mean±SE from ten mice in each group (** *P*<0.0002).

The in vivo role of CCL20 in cancer development is not clear. To determine the role of CCL20 in CXCR4-dependent and -independent tumor development *in vivo*, a tumor xenograft model was used. Human mock-transfected and CCL20-overexpressing PC3 cells were injected subcutaneously into SCID/bg mice. For *in vivo* experiments, we chose to use PC3-CCL20 clones 10 and 30, since these clones demonstrated an increased proliferation rate in culture, and produced either comparable levels of CCL20 (100, and 320 pg/ml) to PC3-CXCR4.5. Mice injected with PC3-CCL20.30 cells developed larger tumors as measured by an increase in size compared to mice injected with mock-transfected PC3 cells (Fig. 4A). Moreover, tumors produced by PC3-CCL20.30 cells were more vascularized and invasive to the neighboring tissues (muscle and dermis) (Fig. 4B). These findings were confirmed by H&E-stained tissue sections of xenograft tumors. Histological analysis of PC-CCL20 tumors demonstrated an invasion of tumors to adjacent muscle tissue, development of necrosis that can be associated with rapid tumor growth, and massive aberrant vascularization of tumors. In contrast, as was previously described, PC3-mock tumors were encapsulated, non-invasive and no aberrant blood vessels were present (Fig. 4B). Mice injected with PC3-CCL20.10 cells also developed larger tumors as measured by an increase in size and weight compared to mice injected with mock-transfected PC3 cells. However, the difference between PC3-CCL20.10 and the parental cells were smaller (Fig. 4A).

Both PC3-CCL20.30 as well as the parental PC3 cells developed a necrotic core, while tumors that overexpressed CCL20 continued to grow beyond this point, suggesting that CCL20 may facilitate angiogenesis in tumors. We studied this hypothesis by comparing the number of blood vessels in histological sections as well as by using an in vivo intra-tumoral vessel functionality MRI based assay in the CCL20 tumors (PC3-CCL20.30) versus that in control tumors (PC3-mock). Macroscopic assessment of tumors overexpressing CCL20 revealed increased vascularization as compared to control tumors (Fig. 4B). To complement the data obtained from histology on blood vessel density, vessel functionality (Δ*S*o_2_) was measured by fMRI to study the actual in vivo perfusion of the tumor and the oxygen delivery efficiency into the tumor mass. Functionality of the vasculature was derived from GE images acquired during inhalation of air-CO_2_ and carbogen (95% oxygen+5% CO_2_) [26] in mice implanted with PC3-mock cells or with PC3-CCL20.30 cells. Interestingly, MRI analysis showed that Δ*S*o_2_ values from PC3-CCL20.30 tumors were significantly higher (Fig. 4C). Δ*S*o_2_ maps derived on day 41 showed enhanced tumor vascularity in tumors produced by PC3-CCL20.30 vs. control tumors that had very low Δ*S*o_2_ values. We left the control tumors to grow for an additional month in order to let them reach similar size. However, even on day 71, they were significantly less vascularized. While functional vessels were observed at the center of tumors overexpressing CCL20, no functional vessels were observed at the center of the control tumors and only on the borders of these tumors. The mean±SD values of Δ*S*o_2_ were calculated from a region of interest containing the whole tumor, and normalized to contra-lateral muscle, pooling data from 9 mice from the PC3-CCL20.30 group and 5 mice from the PC3-mock group (four slices/mouse; Fig. 4C, p<0.001). These results suggest that high expression of CCL20 results in early neovascularization of the tumors while in control tumors, the development of necrosis was mediated by poor perfusion.

### Neutralization of CCL20 inhibits the CCL20 and CXCR4-dependent growth of various tumor cells in vivo

Having established the role of CCL20 in cancer development *in vivo*, we evaluated the effect of neutralizing antibodies to human CCL20 on the growth of CCL20-and CXCR4 overexpressing PC3 cells. First, we tested the ability of anti-CCL20 antibodies to neutralize the CCL20-induced adhesion *in vitro* of PC3-CCL20.30 cells to collagen I. The presence of monoclonal anti-human CCL20 antibodies abolished the adhesion of PC3-CCL20.30 cells to collagen I in response to CCL20 stimulation (Fig. 4D).

Next, we assessed the *in vivo* potential of neutralizing anti-CCL20 antibodies. PC3-CCL20.30 cells were injected subcutaneously into SCID/bg mice. Twenty-four hours after cell injection, mice started to get treatment with subcutaneous injections of anti-human CCL20 antibody or isotype control, 20 µg of antibody per injection, three times a week, during four weeks. A significant decrease in tumor growth was observed in anti-CCL20-treated mice (Fig. 4E, F). In the control group, nine out of ten mice developed subcutaneous tumors which rapidly progressed over time. In contrast, in the ani-CCL20-treated group, only five out of ten mice developed tumors, while four of the produced tumors were very small in size (0.2 cm×0.2 cm or 0.1 cm×0.1 cm) and did not progress over time. Moreover, histological evaluation of H&E-stained tissue sections of xenograft tumors demonstrated that neutralizing antibodies to CCL20 inhibited intensive aberrant blood vessel formation and promoted extensive necrotic tissue damage in treated tumors (data not shown).

To confirm the role of CCL20 in the development and progression of CXCR4-overexpressing prostate tumors, we evaluated the effect of neutralizing antibodies to human CCL20 on the growth of CXCR4-overexpressing PC3 cells. PC3-CXCR4.5 cells were injected subcutaneously into SCID/bg mice. Injected animals were treated with the anti-human CCL20 antibody or isotype control according to the same protocol used in mice injected with CCL20-overexpressing cells. Animals treated with anti-CCL20 antibodies demonstrated a delay in tumor appearance – in the control group on day 28, 100% of animals developed visible tumors, while in the anti-CCL20-treated group, only 60% appeared with tumors on the same day. Furthermore, the treatment with anti-CCL20 antibodies inhibited the growth of CXCR4-expressing prostate tumors (Fig. 4G).

It has recently been shown that CCR6 and CCL20 are significantly upregulated in CRC and colorectal liver metastases (CRLM) [27]. To further test the role of CCL20 in the development and progression of colon cancer, we evaluated the effect of neutralizing antibodies to human CCL20 on the growth of HT-29 tumor cells. HT-29 cells were injected subcutaneously into nude mice. Injected animals were treated daily with the anti-human CCL20 antibody or isotype control. Treatment with anti-CCL20 antibodies significantly inhibited the growth and invasion of HT-29 tumors (Fig. 4H). Moreover, antibodies to CCL20 inhibited the invasion of tumor cells into surrounding tissues similarly to the skin and muscle (data not shown). We therefore concluded that neutralizing antibodies to CCL20 suppressed in vivo CXCR4-dependent and independent prostate and colon tumor growth.

### CXCR4 and CCL20 are co expressed in human prostate cancer

**Figure 5:**
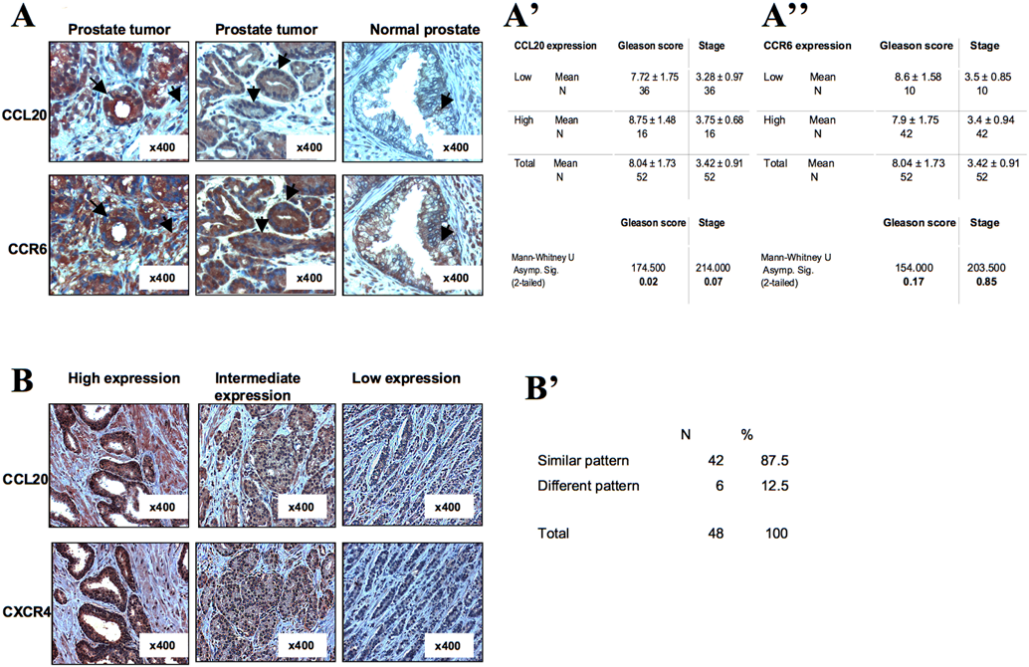
CCL20, CCR6 and CXCR4 expression in prostate cancer cell lines, in primary prostate tumor tissue and in normal prostate tissue. (A) Expression of CCL20 and CCR6 in human prostate cancer tissue and in human normal prostate tissue. Immunohistostaining of prostate cancer and normal specimens using the polyclonal antibody for CCL20 and the monoclonal antibody 140706 for CCR6. Original magnification of ×400 is shown. CCL20 and CCR6 expression was observed in endothelial and fibromuscular cells of prostate samples (signed with black arrows). A commercially available array including 52 samples (CA2) from patients with prostate cancer was stained for CCL20 (A′) and CCR6 (A″). Expression was scored on two levels: low or negative expression, and high expression. Statistical analysis of the immunohistochemical staining was performed using two-tailed Mann-Whitney test. (B) Comnmon expression pattern of CCL20 and CXCR4 in human prostate cancer. Immunohistostaining of prostate cancer specimens using the polyclonal antibody for CCL20 and the monoclonal antibody 12G5 for CXCR4. Original magnification of ×400 is shown. CCL20 and CXCR4 expression was observed in endothelial and fibromuscular cells of prostate samples. (B′) A commercially available array including 48 samples (CA3) from patients with prostate cancer was stained for CCL20 and CXCR4. Expression was scored on three levels: low, intermediate, and high expression. Samples that demonstrated the same scoring in CCL20 and CXCR4 expression levels were classified as having similar expression pattern. Samples where scores in CCL20 and CXCR4 expression differed in one level or more were classified as different expression patterns.

In order to further test the relevance of *in vivo* role of CCL20/CCR6 axis in prostate cancer development, we first evaluated the expression of CCL20 and CCR6 in human prostate cancer tissues with the use of commercially available array of 52 paraffin-embedded prostate sections from patients with advanced prostate cancer. All specimens were graded using pathologic stage and the Gleason score system. The immunohistochemical staining revealed that the majority of tumor samples (50 out of 52, 96%) expressed the CCR6 receptor at heterogeneous levels. Whereas the ligand for CCR6, CCL20, was expressed in 34 out of 52 tumor samples (65.4%). The CCL20 and CCR6 staining were located mostly in epithelial and fibromuscular stromal cells (Fig. 5A). The majority of tumor samples that expressed CCL20 co-expressed CCR6. In contrast, normal human prostate tissue samples expressed very low levels of CCL20 and CCR6 (Fig. 5A). Out of 34 CCL20-positive sections, 16 samples were highly positive for CCL20. Average Gleason score in samples with high CCL20 expression was 8.75±1.48 (n = 16) versus 7.72±1.75 (n = 36) in samples with low or negative CCL20 expression (p = 0.02) (Fig. 5A′). Average stage in samples with high CCL20 expression was 3.28±0.97 (n = 16) versus 3.75±0.68 (n = 36) in samples with low or negative CCL20 expression (p< = 0.07) (Fig. 5A′). No prevalence in high levels of CCR6 in progressive stage IV sections was detected (Fig. 5A″). These results demonstrate that high CCL20 expression correlates with high Gleason score (e.g., tumor grade) and higher staging of the disease in this array.

To further study the expression pattern of CCL20 and CCR6 in prostate tumors, we stained additional specimens from 44 primary tumors. All tissue sections were obtained from the Pathology Department of the Hadassah Hebrew University Medical Center. The average Gleason score in these patients was significantly lower than in the commercial array, 5.77 versus 8.04 respectively. Similar to the commercial array CCL20 was expressed in 70.5% of the tumors and CCR6 was expressed in 95.5% of the tumors sections. However, in contrast to the commercial array no significant correlation between CCL20 expression and Gleason score (e.g., tumor grade) and staging was found. These results suggest that CCL20 and CCR6 are commonly expressed in prostate cancer tumors; however, more studies are needed to demonstrate their diagnostic significance.

In order further test the possible interaction between CXCR4 receptor and CCL20 chemokine in prostate cancer, we evaluated the coexpression of CCL20 and CXCR4 in human prostate cancer tissues with the use of commercially available additional array of 48 paraffin-embedded prostate sections from patients with advanced prostate cancer. Similar to our previous [17] and current stud and the all samples in this array expressed the CXCR4 receptor and CCL20 chemokine at heterogeneous levels. Expression level of CXCR4 and CCL20 was determined by scoring the staining intensity as low, intermediate or high. Co-expression of CXCR4 and CCL20 in the same cancer cells was observed (Fig. 5B). Moreover, similar expression level of CXCR4 and CCL20 was observed on the majority of prostate tumor samples (38 out of 48 samples, 79.5%) (Fig. 5B, 5B′). Four additional samples demonstrated close expression pattern, and only six samples out of 48 (12.5%) demonstrated different expression levels of CXCR4 and CCL20. These results suggest that in human prostate cancer CCR6, CCL20, and CXCR4 are commonly overexpressed and that there is a correlation between CXCR4 and CCL20 expression. More studies are needed to determine the potential diagnostic role for CCL20 and CCR6.

Altogether, our findings for the first time provide evidence for existence of a putative CXCR4/CCL20 interaction that could be involved in cancer development processes. Furthermore, our data suggest an in vivo role of the chemokine CCL20 in CXCR4 dependent and independent regulation of cancer growth and point to CCR6 and CCL20 as novel therapeutic targets in cancer.

## Discussion

Recent studies have revealed the critical multifunctional role of CXCR4-CXCL12 in cancer progression. Here we show that CXCR4 up-regulates the expression of the chemokine CCL20 in various cancer cells, and that CCL20 in turn promotes in an autocrine manner the survival, proliferation and adhesion of cancer cells in vitro and enhances xenograft tumor growth, vascularization and invasion in vivo. CCL20 (also known as macrophage inflammatory protein-3α (MIP3α); liver and activation-regulated chemokine (LARC) or exodus-1) is a 9 kDa CC-type chemokine, which is expressed constitutively at low levels by keratinocytes in epidermal layers of skin [28] intestinal mucosa [29], liver [30], epithelial crypts of tonsils [31], as well as in the epithelium of Peyer's patches in the intestine [32]. While there is redundancy in the human chemokine network, CCL20 is the unique chemokine ligand of its receptor CCR6. Expression of CCL20 is strongly enhanced by pro-inflammatory stimuli, such as IL-1β, TNF-α, and LPS [33], [34]. CCL20 selectively attracts CCR6-expressing natural killer cells, memory T cells, TH17 cells, and immature dendritic cells (DC) to the sites of inflammation to encounter the invading pathogens.

Recent studies suggested that CCL20 is an important player in the tumor chemokine network and similarly to other chemokines, CCL20 may promote cancer progression by two mechanisms: acting directly as a growth factor on cancer cells or establishing a microenvironment that suppresses specific anticancer responses. CCL20 expression has been described in a variety of human neoplasms, including colorectal, lung, pancreatic and breast human adenocarcinomas, malignant glioma, leukemia, lymphoma and melanoma However, the role of CCL20 in cancer development remains controversial. Since CCL20 is a potent chemoattractant for immature DCs, the most powerful antigen-presenting cells, it seems logical to attract iDCs to the tumor site to induce antitumor immune response. According to this approach, Fushimi et al., demonstrated in a mouse model that intratumor injection of adenovirus-mediated gene transfer of CCL20 could suppress tumor growth [35]. On the other hand, there is a growing body of evidence supporting the hypothesis that CCL20 production by cancer cells promotes tumor growth and invasiveness. Contrary to the results of Fushimi et al., other groups of investigators have shown that transfection of rodent tumor cell line with CCL20 enhances tumor growth and decreases immunogenicity, despite the attraction of iDCs to the tumors [36]. In their model, as in human breast carcinomas which secrete high levels of CCL20 [37], attracted DCs to the tumor site did not mature. These may serve as one of the tumor escape mechanisms by which attracted immature DCs poorly stimulate T-cell antitumor response, and moreover can activate regulatory T cells which suppress specific anti-tumor immune responses.

In our experimental models, we chose to evaluate the involvement of CCL20 in prostate and colon tumor growth, vascularization and invasiveness. Our results match the observations of Kleef et al., who demonstrated that CCL20 is overexpressed in human pancreatic carcinoma cells and in infiltrating macrophages adjacent to tumors, and stimulates the growth and invasion of neoplastic cells [38]. Up- regulation of CCL20 was demonstrated in human hepatocellular carcinoma tissues, and CCL20 expression was found to correlate with tumor grade [39]. Furthermore, we found that CXCR4, CCL20 and CCR6 are over- and coexpressed in human prostate cancer specimens (Fig. 5). In support of our findings it was recently published that the expression levels of CCR6 in prostate cancer are associated with clinical and pathologic features of more advanced and aggressive prostate cancer [40]. Here we show that, CCL20 promotes the growth and adhesion of CCR6-expressing cancer cell lines. Moreover, overexpression of CCL20 or CXCR4 in prostate and colon cancer cells promotes tumor growth that was neutralized with anti-CCL20 antibodies. These findings strongly emphasize the potential co-involvement of CCL20 and CXCR4 in cancer development.

Altogether, our findings provide evidence for the existence of a putative CXCR4/CCL20 interaction that could be involved in cancer development processes. Furthermore, our data suggest a role of the chemokine CCL20 in CXCR4-dependent and independent regulation of cancer growth and point to CCR6 and CCL20 as novel therapeutic targets in cancer.

## Materials and Methods

### Cell culture and transduction of cell lines and cell clones

The following human cell lines were used in the study: prostate cell lines PC3 (CRL-1435), LNCaP (CRL-10995), 22Rv1 (CRL-2505), DU145 (HTB-81); acute promyelocytic leukemia cell lines NB4, HL-60 (CCL-240) and colon carcinoma cell line HT-29 (HTB-38). PC3 cell line stably overexpressing CCL20 (PC3-CCL20) was produced by transfection of PC3 cells pcDNA3-CCL20 construct. PC3-CCL20 single-cell clones were produced by limited dilutions. The level of secreted CCL20 protein was analyzed in supernatant of PC3-CCL20 clones using ELISA kit (R&D Systems, Minneapolis, MN). PC3-CXCR4 single-cell clones were produced by limited dilutions.

### RNA extraction and reverse transcription ad Semi-quantitative and Real-Time PCR

Total RNA was extracted from prostate and leukemic cell lines using TRIzol reagent (Invitrogen Life Technologies). For cDNA synthesis, 2.5 microgram of total RNA were reverse-transcribed and the following primer pairs were used for PCR: β-actin sense 5′ - CCCTGGACTTCGAGCAAGAG′ - 3′, antisense 5′ - TCTCCTTCTGCATCCTGTCG - 3′; CCL20 sense 5′ – ATGTGCTGTACCAAGAGTTT - 3′, antisense 5′ - CAAGTCTGTTTTGGATTTGC - 3′; CCR6 sense 5′ – CCATTCTGGGCAGTGAGTCA - 3′, antisense 5′ - AGCAGCATCCCGCAGTTAA - 3′; CXCR4 sense 5′ - AGCTGTTGGCTGAAAAGGTGGTCTATG - 3′, antisense 5′ - GCGCTTCTGGTGGCCCTTGGAGTGTG - 3′; CXCL12 sense 5′ - ATGAACGCCAAGGTCGTGGTCG - 3′, antisense 5′ - TGTTGTTGTTCTTCAGCCG - 3′. CCL20 quantitative PCR assay containing the primers and probe mix was purchased from Applied Biosystems, Foster City, CA, All reactions were run in triplicates using ABI Prism 7700 Sequence Detector System (Applied Biosystems). Gene expression of CCL20 gene was analyzed in relation to the levels of the housekeeping β-actin gene.

### Cell proliferation assay

The effect of CCL20 on the viability of PC3, PC3-CXCR4.5, PC3-CCL20 clones and leukemia NB4 and HL60 cells was studied. In brief, PC3 and PC3-CXCR4.5 cells were seeded at 2×10^4^ cells/1 ml per well into a 24-well plate in medium supplemented with 0.1% FCS with or without various concentrations of CCL20 (PeproTech EC, London, UK). Following three days, the medium with or without CCL20 was renewed. On day six, the attached cells were harvested, stained with propidium iodide (Sigma, St. Louis, MO), and the number of viable cells was determined using FACS analysis. Optionally, PC3 and PC3-CXCR4.5 cells were labeled with 5-bromo-2-deoxyuridine (BrdU) (Sigma, St. Louis, MO) at a concentration of 10 µM during the last 16 hours of incubation and processed for BrdU detection using specific anti-BrdU antibody (eBioscience) and FACS analysis.

### Cell adhesion assay

Prostate cancer cells (PC3, PC3-CXCR4.5 and PC3-CCL20 clones), leukemia NB4 and HL60 cells, and colon cancer cells HT-29 (1×10^5^/500 µl) were allowed to adhere to 10 µg/ml fibronectin-coated or collagen type I-coated 24-well plates for 30 minutes at 37°C in serum-free RPMI supplemented with 0.1% bovine serum albumin (BSA). Non-adherent cells were washed twice with cold PBS. Adherent cells were collected in 300 µl FACS buffer (PBS× 1+0.1% BSA+0.01% NaNO_3_) with 5 mM EDTA and counted by FACScalibur (Becton Dickinson Immunocytometry Systems).

### ELISA assay

Prostate cancer cells (PC3, PC3-CXCR4.5 and PC3-CCL20 clones), leukemia cells NB4 and HL60 and colon cancer cells HT-29 were seeded into a 12-well plate at 2×10^5^/1 ml of medium per well with various concentrations of CXCL12 (5–1,000 ng/ml) (PeproTech EC, London, UK) or PTX (5 µg/ml) (List Biological Laboratories, Campbell, CA, USA). The cells were incubated for 48 hours, supernatants were collected and CCL20 protein levels were determined using ELISA kit (R&D Systems, Minneapolis, MN). For intracellular CCL20 detection, cells were harvested and lysed with lysis buffer containing 50 mM Tris-HCl, pH 7.6, 150 mM NaCl, 5 mM EDTA pH 8.0, 0.5% NP40 and protease inhibitors cocktail (Roche Diagnostics, Mannheim, Germany). Cell lysates were centrifuged at 14,000 *g* for 20 minutes at 4°C and equal amounts of protein extracts were applied for ELISA analysis.

### Flow cytometric analysis

In order to characterize the expression levels of chemokine receptors CXCR4 and CCR6 on cancer cell lines, the cells were stained with human specific direct-labeled antibodies and analyzed by FACScalibur (Becton Dickinson Immunocytometry Systems), using CellQuest software. For CXCR4 expression analysis, anti-human CXCR4 monoclonal antibody, clone 12G5 (R&D Systems, Minneapolis, MN) or polyclonal anti-N-terminus antibody (Chemicon International, Temecula, CA, USA) were used. For CCR6, anti-human CCR6 monoclonal antibody, clone 53103.11 (R&D Systems, Minneapolis, MN) was used.

### RNA interference

The Dicer-substrate siRNA duplexes of CXCR4 (NCBI accession number NM_003467): sense 5′ - UAAAAUCUUCCUGCCCACCAUCUAC - 3′, antisense 5′ – GUAGAUGGUGGGCAGGAAGAUUUUAUU - 3′ were purchased from IDT, Coralville, IA. The non-specific siRNA duplexes were used as a control. PC3-CXCR4.5 cells were transfected with 200 nmol/L siRNA in serum-free medium using Oligofectamine reagent (Invitrogen, Carlsbad, CA, USA) according to the manufacturer's instructions.

### Establishment of tumor xenografts

Nude and SCID/beige mice (C.B -17/IcrHsd-SCID-bg) were maintained under defined flora conditions at the Hebrew University Pathogen-Free Animal Facillity. All experiments were approved by the Animal Care Committee of the Hebrew University. Prostate cancer cell lines (PC3, PC3-CXCR4.5, PC3-CCL20.30 and PC3-CCL20.10) and HT 29 cells were grown to 80% confluence, harvested, and injected subcutaneously in the flank of mice (5×10^6^/mouse). Once palpable, tumors were measured and tumor size (width×length) was calculated. For the neutralizing experiments, mice were treated with subcutaneous injections of monoclonal anti-human CCL20 antibody (MAB360, R&D Systems, Minneapolis, MN) or control IgG1 antibody. At the end of the experiments, animals were sacrificed, tumors were harvested, measured and weighted. MRI analysis of tumor growth and blood vessel functionality and maturation were done as previously described (18).

### Immunohistochemistry and scoring

Two different commercial prostate tumor tissue microarrays were used: CA2 array included 52 prostate cancer tissue sections and CA3 array included 48 prostate cancer tissue sections (SuperBioChip Lab). In addition tissue samples of primary prostate tumors from 41 patients, 2 were collected from the archives of the Pathology Department of the Hadassah Medical Organization, Jerusalem, Israel. Formalin-fixed, paraffin-embedded tissue samples were initially dewaxed, rehydrated, treated with EDTA buffer and blocked with CAS blocking reagent (Zymed Laboratories, San Francisco, CA, USA) for 30 minutes in room temperature. Samples were then incubated overnight at 4°C in a humidified chamber with anti-human CCL20 polyclonal antibody (PeproTech EC, London, UK), diluted to final concentration 20 µg/ml, or alternatively with anti-human CCR6 monoclonal antibody (R&D Systems, Minneapolis, MN) diluted to final concentration 10 µg/ml, or with anti-human CXCR4 monoclonal antibody, clone 12G5 (R&D Systems, Minneapolis, MN) diluted to final concentration 10 µg/ml. Next, the sections stained for CCL20 were incubated with diluted 1∶1000 biotinylated goat-anti-rabbit antibody (Jackson ImmunoResearch), for 30 minutes at room temperature and thereafter with horseradish peroxidase -conjugated streptavidin (Zymed Laboratories, San Francisco, CA, USA) for 30 minutes at room temperature. The sections stained for CCR6 or CXCR4 were incubated with secondary anti-mouse horseradish peroxidase-conjugated antibody (DakoCytomation, Glostrup, Denmark) for 30 minutes at room temperature. 3-amino-9-ethylcarbazole (AEC) was used for color development, and sections were counterstained with hematoxylin.

Analysis of CCL20 and CCR6 expression was determined by scoring the staining intensity as negative, weak or strong by two independent investigators. Scoring was performed blindly, without knowledge of overall Gleason score or tumor pathologic stage.

### Statistical analysis

Data are presented as means±SD or ±SE. Statistical comparison of means was performed by a two-tailed unpaired Student's *t* test. Differences with a P<0.05 were determined as statistically significant.
